# The prevalence of patients suffering from chronic spontaneous urticaria, in whom omalizumab cannot be stopped even after six years

**DOI:** 10.3389/falgy.2024.1464466

**Published:** 2024-10-02

**Authors:** V. Schichter-Konfino, R. Mubariki, E. Toubi, Z. Vadasz

**Affiliations:** ^1^ Hillel Yaffe Medical Center-Hadera, The Rappaport Faculty of Medicine, Technion, Haifa, Israel; ^2^ Bnai-Zion Medical Center, The Rappaport Faculty of Medicine, Technion, Haifa, Israel; ^3^ The Holy Family Hospital- The Azrieli Faculty of Medicine, Nazareth, Israel

**Keywords:** omalizumab, CSU, IgE, anti-TPO, asthma, autoimmunity

## Abstract

**Background:**

Omalizumab (OMA) was the first FDA-approved biological drug for severe chronic spontaneous urticaria (CSU), and until today is the only beneficial and truly safe one. The objectives were: To assess the prevalence of CSU patients in whom OMA cannot be stopped over time. We also asked if biomarkers (e.g., anti-TPO antibodies and total IgE) could assist in anticipating this issue.

**Methods:**

We used our prospective registry of 93 patients, which included CSU disease duration, the onset of OMA treatment, Urticaria Activity Score (UAS7) during follow-up, co-morbidities, serum IgE levels and the presence of anti-TPO antibodies. Finally, we assessed the response to OMA during a period of six years.

**Results:**

Out of the 93 treated CSU patients, OMA was stopped in ten patients after six months being defined as failures. In another ten patients, OMA was discontinued after 2–4 years of therapy, achieving a remission. Seventy-three patients are still treated between 2 and 6 years, having different degrees of response. Of these, in thirty-eight (52%) patients, we could not stop OMA even after six years due to CSU relapses. The prevalence of lower serum IgE levels and anti-TPO antibody positivity was significantly higher in CSU patients in whom OMA could not be stopped.

**Conclusion:**

This is the first study where OMA-treated CSU patients were followed up to six years. In half of them, long-term therapy of six years is still required.

## Highlights

• Omalizumab is required for more than 6 years in half of continuously CSU treated patients.
• Omalizumab can be discontinued in only 10% of treated patients after two-four years without experiencing any relapse.
• Low levels of total IgE and anti-TPO antibodies are biomarkers for anticipating the need for long-term omalizumab treatment.

## Introduction

Until more than a decade ago, cyclosporine A (CsA) was the only effective drug for treating severe cases of CSU that were refractory to high doses of anti-histamines and almost always dependent on short courses of corticosteroids (1, 2). In 2011, the beneficial and safe effect of omalizumab (OMA; an IgG-anti-IgE antibody) in unresponsive CSU patients was reported in a randomized placebo-controlled study (3). The publication of later studies in which OMA was shown to be highly beneficial in severe cases of CSU, was a crucial step in establishing OMA as the first successful biological drug for CSU (4, 5). Following these reports, a consensus conference held in November 2012 in Berlin, was the first to include OMA as an optional drug for treating severe CSU. The 2013 revision and update of this consensus conference became the most accepted guideline for the diagnosis and treatment of CSU (6). In 2014, the FDA approved OMA to be a useful drug for severe and refractory cases of CSU. Shortly after that, the up-dated guidelines for treating CSU including the usage of OMA were authorized in many countries including Israel in 2015. During the last five years, most studies on OMA and CSU focused on its efficacy, safety, and predictors of response. The definition of clinical responses and therapeutic outcomes to OMA is different and is not consistent between clinical trials, and real-life studies. In this respect, the percentage of complete response, partial response and failure to respond to OMA therapy is dependent on how long follow-up was maintained, and which of the accepted tools for evaluating the response [Urticaria Activity Score (UAS7) or Urticaria Control Test (UCT)] were used (7). When CSU patients remain symptomatic at 300 mg for six months and require almost daily antihistamine treatment, up dosing to 450 mg OMA frequently achieves an additional decrease in the UAS7 score and a further improvement in the quality of life of these patients. Higher doses of OMA are effective and safe, and can be maintained for long periods of time (8). One of the topics of recent studies concerning OMA and CSU is how to predict the response to licensed doses of 300 mg. The literature suggested many predictive factors including, autoimmune thyroid disease, and CSU duration before the initiation of OMA, angioedema, and baseline levels of total IgE (9). During the last decade, OMA has remained the most advanced and efficient biological therapy for severe CSU patients, who are unresponsive to high doses of antihistamines and short courses of steroids. In this respect, the questions we want to answer are: what is the percentage of CSU patients in whom OMA therapy failed? How many patients can stop therapy without experiencing any relapse? Finally and mostly undefined, is the percentage of patients in whom OMA could not be stopped even after six years? Can all this be anticipated by using relevant biomarkers? Most studies summarized a follow-up of only 12 months of OMA therapy, causing many of the above-mentioned questions to remain unclear. To assess all the above questions, we went through our registry of 93 CSU OMA- treated patients from our three outpatient clinics in Israel. We started this prospective registry in 2013 and all patients were followed until March 2023.

## Methods

### Patients population

The study included ninety-three CSU patients (F = 62, M = 31, mean age 48 ± 3.6), in whom OMA was given after failing standard therapy, including high doses of antihistamines and few short courses of steroids. Our registry included the following information: CSU disease duration, the onset of OMA treatment, UAS7 during the whole period of follow-up, the response to OMA therapy and when higher dosage (450 mg) was required. It also included biomarkers such as serum total IgE levels (low IgE levels <40 IU/ml and high levels >100 IU/ml), anti-TPO IgG antibodies, and the co-presence of co-morbidities such as autoimmune disorders (e.g., Hashimoto thyroiditis and Celiac diseases) and atopic diseases. Finally, it included information about how frequently we could open a gap of five to six weeks between two treatments, and whether OMA could be stopped at some point. The response to OMA was assessed as follows: (a) Complete response when UAS7 is reduced up to 0–1 points. (b) Good response was defined when UAS7 remained in the range of 2–8 points. (c) Fair response was defined when OMA did not achieve a reduction of 50% of baseline UAS7 and requiring additional treatment. (d) Failure of therapy was defined when patients remained active and completely unsatisfied and UAS7 remained higher than 75% of baseline.

### Statistical analysis

Statistical analysis was conducted using one way ANOVA, followed by Tukey *Post-Hoc* test.

## Results

Mean disease duration during follow-up was 42 months (range 10–72 months). All patients were highly active having a mean UAS7 of 34 before starting OMA. The CSU disease duration was longer among fair responders and failures. The onset of OMA treatment was similar in all groups (treatment was started in all patients between 1.7–2.5 years after the onset of CSU) (see Table 1). The delay in starting OMA was because CSU patients were first treated in general clinics and only later were referred to our specialized clinics. Out of 93 CSU patients, ten patients were defined as failures after failing a higher dosage of 450 mg and therefore OMA was stopped after six months of the higher dose. Eighty-three patients were maintained on OMA during the whole period of follow up, achieving different reductions of UAS7. A complete response was achieved in 23 (24.7%) of treated patients, a good response was maintained in 38 (40.8%), 22 (23.6%) were defined as fair responders and10 patients were defined as failures (10.7%). In 53 patients (64%), OMA dosage was maintained at 300 mg whereas in 30 patients (36%) the up dosing of OMA to 450 mg was required being unable to achieve at least a good response during 12 months. Out of the 23 complete responders, we could stop OMA treatment in ten patients (43.5%) after 2–4 years, remaining in full remission during one year of follow-up, thus considered as cured. Out of the remaining 73 CSU patients in whom OMA is continued, 13 are complete responders (17.8%), 38 are good (52%) and 22 are fair responders (30.1%). Of these, OMA is maintained over 6 years in 38 patients (52%), in whom it is impossible to stop treatment. Of importance to notice that, levels of total IgE and anti-TPO were not available in all patients.

**Table 1:** The characteristics of OMA treated CSU patients.

	CSU disease duration (years)	Start of treatment (years)	IgE >100	IgE <40	anti-TPO	Asthma	Autoimmunity
Complete response (*n* = 23)	2.6	1.7	10/19 (52.6%)	9/19 (47.4%)	3/20 (15%)	34.7%	0%
Good response(*n* = 38)	3	1.9	11/29 (37.9%)	18/29 (62.1%)	5/26 (19.2%)	13.1%	5.2%
Fair response (*n* = 22)	3.6	2.3	2/16 (12.5%)	14/16 (87.5%)	7/19 (36.8%)	13.6	4.5%
Failure (*n* = 10)	4	2.5	0/10 (0%)	10/10 (100%)	6/10 (60%)	10%	30%

### Long-term OMA therapy

Out of the 73 CSU patients that are maintained on OMA treatment, 38 (52%) patients are still under continuous treated with OMA up to 6 years. Of these, eight patients (21%) are complete responders (but repeated attempts to stop therapy failed), twenty-three are good responders (60.5%), and seven (18.4%) are fair responders (yet are satisfied on OMA treatment every four weeks). However, in all of them OMA cannot be stopped. All complete and good responders are maintained on 300 mg and seven fair responders are on 450 mg. In complete responders and in nine of good responders, a gap of 5–6 weeks between two treatments could keep patients sufficiently controlled, but all others had to be treated every four weeks (see Table 2). Focused on CSU patients on long-term OMA therapy, 9/29 patients had high serum levels of total IgE and 20/29 had low levels. In terms of autoimmunity, eight patients are positive to anti-TPO antibodies and two have autoimmune disorders, namely, Celiac disease, and Hashimoto thyroiditis.

**Table 2:** Characteristics of only long term (>6 years) OMA treated patients.

	Complete response	Good response	Fair response
*n* = 38	8/38 (21%)	23/38 (60.5%)	7/38 (18.4%)
Dosage (mg)	300	300	450
Frequency of treatment	Once every 5–6 weeks	40% once every 5–6 weeks	Once every 4 weeks
		60% once every 4 weeks	

### Response to OMA and IgE levels

**Figure 1:**
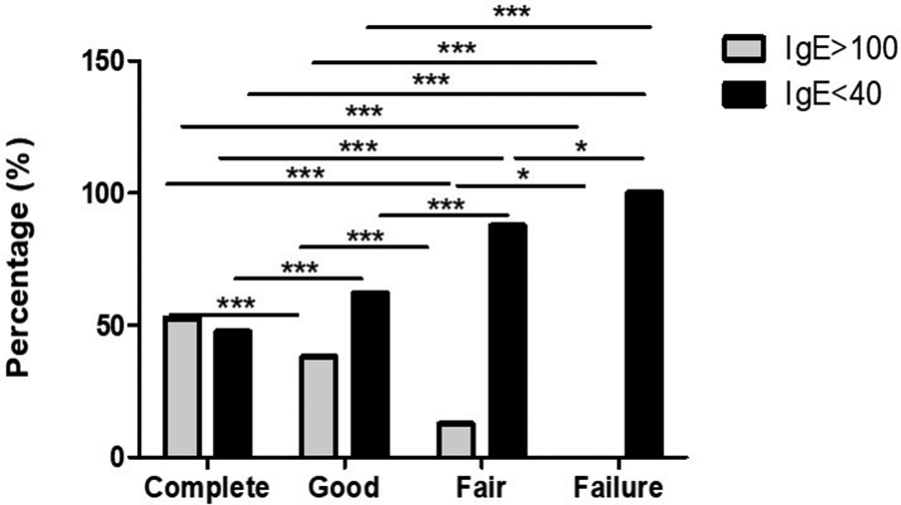
IgE serum level is a good biomarker for assessing the response to omalizumb in CSU. Low IgE levels are predictors of fair response. Significantly, lower IgE levels are recorder in fair and failure responders in comparison to complete and good responders. *p-value <0.05, ***p-value <0.001.

Among complete responder, total IgE levels were assessed in 19 patients. High levels were found in ten (52.6%) and low levels in nine (47.4%). In good responders, total IgE was analyzed in 29 patients. High levels were found in eleven (37.9%) and low in eighteen patients (62.1%). In fair responders, total IgE was analyzed in only sixteen patients. High levels were found in only two (12.5%) and low levels in fourteen (87.5%). Finally, all failures had low levels of total IgE (See Figure 1).

### Response to OMA and anti-TPO antibodies

**Figure 2:**
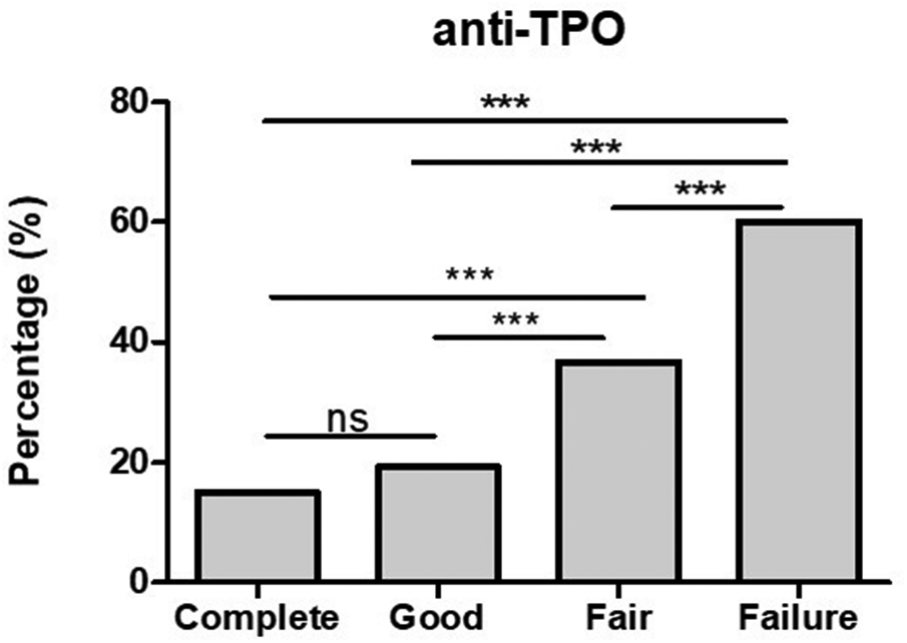
The finding of positive anti-TPO antibodies is significantly higher in fair responders and failures in comparison with good and complete responders. ***p-value <0.001, ns >0.05.

**Figure 3:**
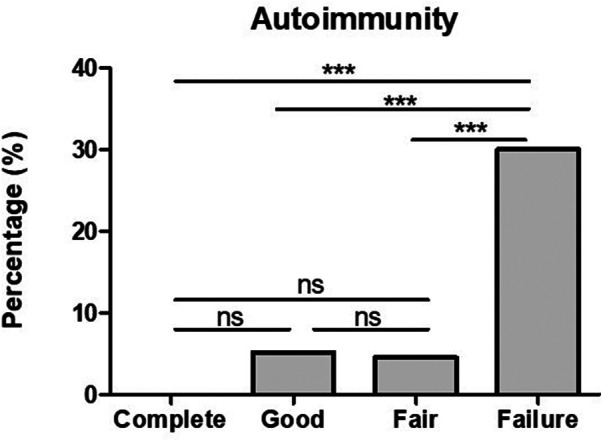
The presence of autoimmunity is significantly higher in CSU patients who failed omalizumab treatment in comparison to those who had a complete, good and fair response. ***p-value <0.001, ns >0.05.

Positive anti-TPO antibodies were found in 3/20 (15%) complete responders assessed, and in 5/26 (19.2%) good responders. However, looking into fair responders and failures anti-TPO was found significantly higher in comparison to complete and good responders (Figure 2). In addition to the finding of positive anti-TPO antibodies, we assessed the presence of autoimmune disorders, specifically, Hashimoto thyroiditis, rheumatoid arthritis, celiac disease, and alopecia all of which were found significantly higher in failures in comparison with complete, good and fair responders (see Figure 3).

### Response to OMA and asthma

**Figure 4:**
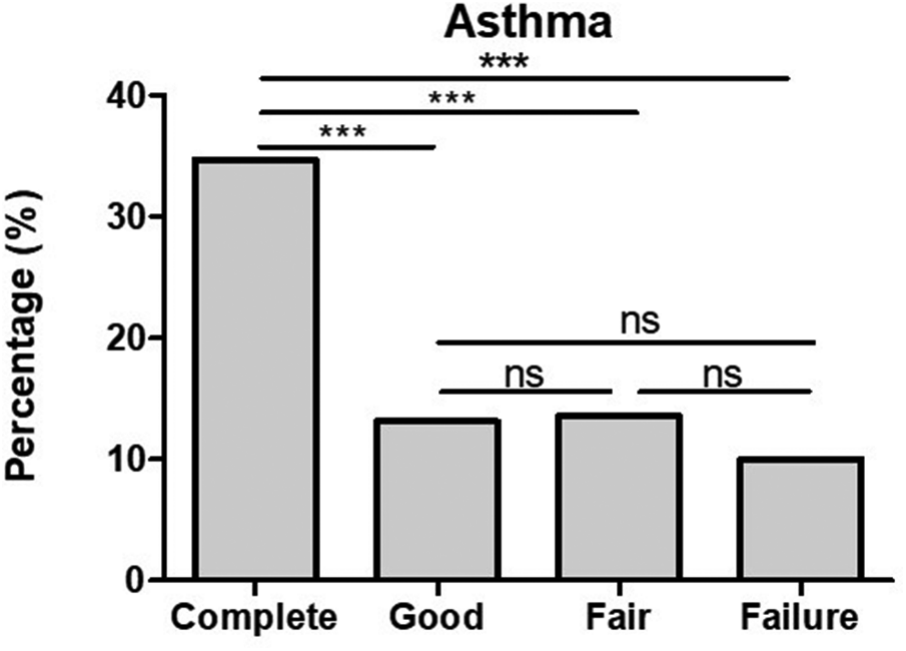
The co-presence of bronchial asthma in CSU patients is significantly higher in complete response in comparison to that in good and fair responders and failures. ***p-value <0.001, ns >0.05.

The co-presence of asthma in complete was significantly higher when compared to those of good and fair responders as well as failures (see Figure 4).

## Discussion

The issue of CSU duration is variably reported in hundreds of studies. Looking into ten recently published studies, and relevant conference proceedings, we found that CSU lasted up to five years in 34%–45% of patients. In many, it may last longer. Most of them suffer from severe CSU and require intensive follow-up and treatment (10). For ten years, OMA has remained the only FDA approved biological drug found to be a safe and highly efficient therapy for severe CSU. The beneficial effect of OMA and its safety over time have been well established in many studies, but little is known about the need for the continuation of this treatment over the long term. Until recently, most studies evaluated OMA efficacy and treatment duration with a follow- up period of no more than 12 months. In an early study, OMA treatment for 24 weeks achieved a complete response in only 35.8% of patients and a good response in 51% (11). In a recent study, 60% were defined as complete and good responders, 25% as partial responders, and 15% were defined as non-responders. In 27% of patients OMA was stopped, but in 38% of them, CSU relapsed, and treatment was re-started (12). The limitations of these studies were mainly the short follow-up period (between 6 and 12 months) and the inconsistencies in assessing the methods of response. Thus, the question of the percentage of patients in whom OMA treatment can be stopped at some point, keeping CSU in almost full remission, or determining percentage of CSU patients who require long-term treatment (more than five years) are still missing. An additional issue is whether the presence of co-morbidities such as asthma and biomarkers such as serum levels of total IgE or anti-TPO antibodies are useful in predicting the issues discussed above. This is the first study in which we are using our prospective registry and long-term prospective follow-up, to try to answer some of the open questions mentioned above. Similarly, to previous studies (13), in our current study, a complete response was associated with high serum levels of total IgE and the high prevalence of asthma. However, Low IgE levels and autoimmunity are associated with a faire response and failure to therapy. Out of the 93 patients in whom OMA was maintained, only ten patients were able to stop treatment, keeping CSU in almost full remission for more than one year. However, our main interesting finding is that in 38 (52%) out of the continuously treated patients, OMA could not be stopped even after six years. In these cases, stopping OMA was followed by a CSU relapse after 3–5 months. Looking into this group, we were able to find a higher percentage of autoimmunity, namely, the presence of autoimmune disorders and anti-TPO antibodies and a higher prevalence of low levels of IgE. The duration of CSU before the initiation of OMA may anticipate the outcome of these patients. We noticed that fair responders and failures had longer CSU duration before OMA was started. In our registry, most CSU patients were put on OMA treatment a long time after the onset of the disease, this was due to the fact that in our country, many CSU patients are long followed in general clinics, before they are referred to specialized clinics. Thus, one may assume that earlier OMA administration may result in a better outcome. Future studies may approve the usage of the above clinical/laboratory biomarkers for predicting the duration of OMA therapy. Finally, future biological therapies (once approved) should be included in CSU guidelines, with the hope of achieving better results.

## Data Availability

The original contributions presented in the study are included in the article/Supplementary Material, further inquiries can be directed to the corresponding author.
